# Targeting cGAS/STING signaling-mediated myeloid immune cell dysfunction in TIME

**DOI:** 10.1186/s12929-023-00942-2

**Published:** 2023-06-28

**Authors:** Vijay Kumar, Caitlin Bauer, John H. Stewart

**Affiliations:** 1 Department of Interdisciplinary Oncology, Stanley S. Scott Cancer Center, School of Medicine, Louisiana State University Health Science Center (LSUHSC), 1700 Tulane Avenue, New Orleans, LA 70012 USA; 2 Louisiana Children’s Medical Center Cancer Center, Stanley S. Scott Cancer Center, School of Medicine, Louisiana State University Health Science Center (LSUHSC), 1700 Tulane Avenue, New Orleans, LA 70012 USA; 3 Surgery, Section of Surgical Oncology, Louisiana State University New Orleans-Louisiana Children’s Medical Center Cancer Center, Louisiana State University Health Science Center (LSUHSC), 1700 Tulane Avenue, New Orleans, LA 70012 USA

**Keywords:** Cancer, cGAS, STING, MIC, Macrophages, DCs, MDSCs, TME, TIME

## Abstract

Myeloid immune cells (MICs) are potent innate immune cells serving as first responders to invading pathogens and internal changes to cellular homeostasis. Cancer is a stage of altered cellular homeostasis that can originate in response to different pathogens, chemical carcinogens, and internal genetic/epigenetic changes. MICs express several pattern recognition receptors (PRRs) on their membranes, cytosol, and organelles, recognizing systemic, tissue, and organ-specific altered homeostasis. cGAS/STING signaling is a cytosolic PRR system for identifying cytosolic double-stranded DNA (dsDNA) in a sequence-independent but size-dependent manner. The longer the cytosolic dsDNA size, the stronger the cGAS/STING signaling activation with increased type 1 interferon (IFN) and NF-κB-dependent cytokines and chemokines’ generation. The present article discusses tumor-supportive changes occurring in the tumor microenvironment (TME) or tumor immune microenvironment (TIME) MICs, specifically emphasizing cGAS/STING signaling-dependent alteration. The article further discusses utilizing MIC-specific cGAS/STING signaling modulation as critical tumor immunotherapy to alter TIME.

## Introduction

The tumor immune microenvironment (TIME) molecular milieu supports chronic inflammation and associated carcinogenesis through immunosuppressive cytokines (TGF-β, IL-10, IL-4, IL-6, IL-13, and IL-33), reactive oxygen species (ROS), reactive nitrogen species (RNS), angiogenic factors [vascular endothelial growth factor (VEGF) and hypoxia-inducible factor (HIF-1α)], and carcinogenic inflammatory pathway stimulating transcription factors (TFs), including nuclear factor-kappa B (NF-κB) and signal transducer and activator of transcription-3 (STAT-3) [1–5]. Tumor-associated macrophages (TAMs), neutrophils (TANs), and myeloid-derived suppressor cells (MDSCs) are major MICs involved in generating tumor-supportive immunosuppressive cytokines, ROS, RNS, and angiogenic factors [6–10]. The interactions between these TFs enhance chronic tumoral inflammation and suppress antitumor immune responses, thereby supporting tumor growth, progression, and metastasis [11]. Furthermore, another TF, interferon regulatory factor 4 (IRF4), is strongly associated with the anti-inflammatory and immunosuppressive M2 macrophage (CD163^+^) phenotype that supports tumor growth [12, 13]. Thus, the immunosuppressive tumor supportive TIME comprises anti-inflammatory myeloid immune cells (MICs), including M2 macrophages, N2 neutrophils, and MDSCs, along with tolerogenic dendritic cells (tDCs), and increased number of regulatory T (T_regs_) and B cells (B_regs_) [14–19].

Macrophages are the most abundant immune cells, comprising ~ 50% of hematopoietic cells in different cancers [20–23]. Recently, a study has indicated that macrophages (12.3% of total cells and 34.1% of immune cells) are the highest number of immune cells in lung cancer or lung adenocarcinoma (LUAD) [13]. The high number of macrophages (CD163^+^ macrophages, non-classical monocytes, and intermediate monocytes) in LUAD is consistent with early findings indicating their crucial protumorigenic role in non-small cell lung cancer (NSCLC) niche [13, 24]. In LUAD, the prevalence of CD163^+^ macrophages strongly correlates with immunosuppressive or immunoregulatory T_regs_. Similarly, brain TIME of glioblastoma (GBM) and brain metastasis (BrM) have a higher number of monocyte-derived macrophages (30.5%) and microglia (9.2%, residential brain macrophages) [25]. LUAD, GBM, and BrM TIME have increased CD163^+^ M2-like macrophages. Different strategies (receptor-ligand interaction, intracellular targeting, epigenetic modification, immunometabolism, angiogenesis, and genetic modification) have been employed to therapeutically target MICs in TME as their accumulation modifies the T cell receptor (TCR)-modified/engineered T cells in different solid cancers [26–30]. For example, several clinical trials targeting MICs broadly in many tumors have reported no results or were terminated earlier due to toxicity issues at different stages [30]. Among these drugs/molecules are modulators of colony-stimulating factor 1 receptor (CSF1R), CC-chemokine receptor 2 (CCR2), CXC-chemokine receptor 2 (CXCR2) and phosphoinositide 3-kinase (PI3K) specifically in MICs [30]. These molecules worked well in single-arm phase-I and small phase-II clinical trials but not in large phase II and III clinical trials as they showed different toxicities. Therefore, we must hunt for novel immune mechanisms involved in MIC dysfunction and their targeting in TIME.

**Fig. 1:**
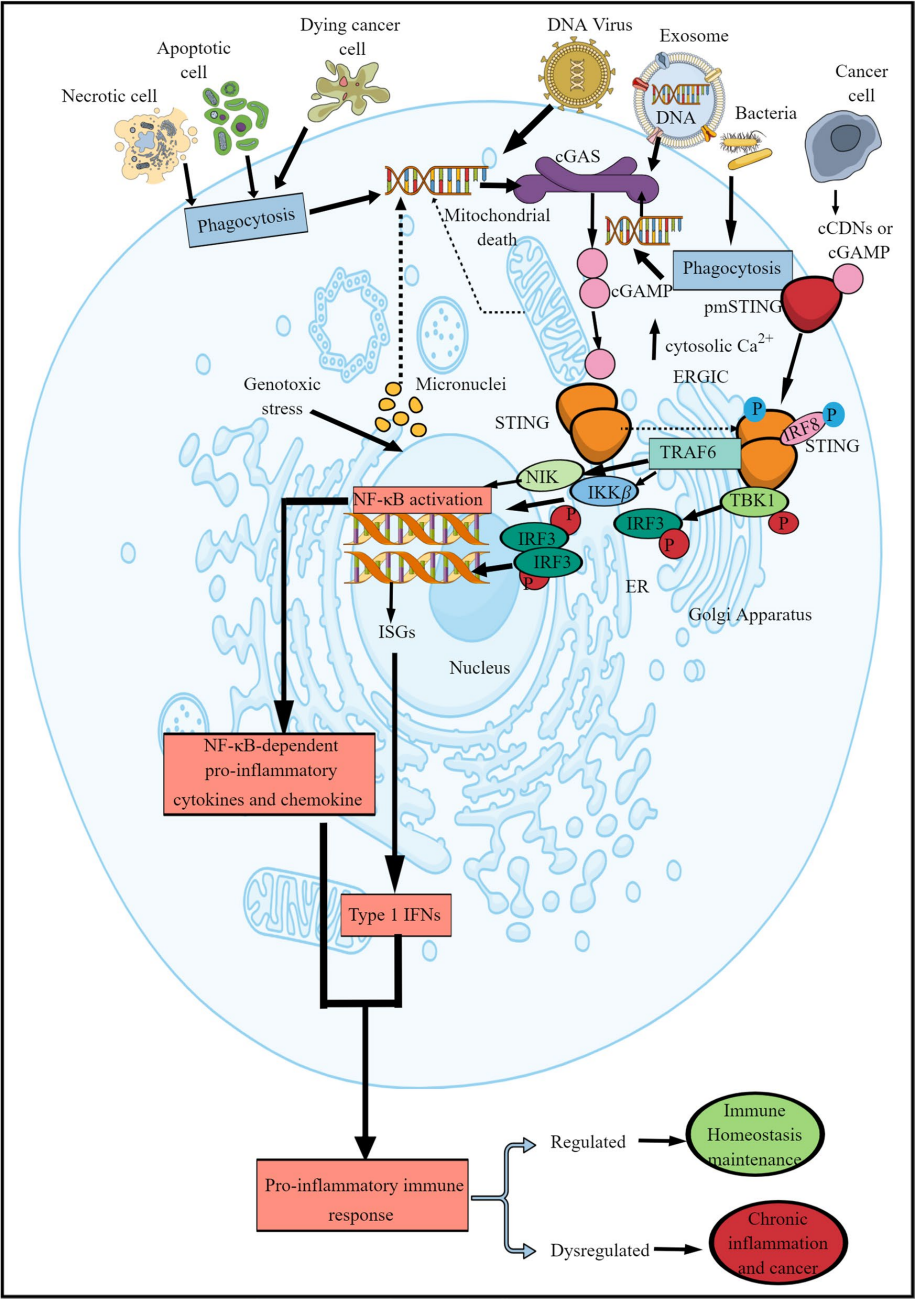
Schematic representation of cGAS/STING signaling in MICs. cGAS is a cytosolic PRR that recognizes and cleaves cytosolic dsDNA into CDN called cGAMP. The cytosolic dsDNA can be self-derived, including the mitochondrial DNA, produced during ER and genotoxic stress and mitochondrial stress and death. Also, MICs can get other cells’ DNA by phagocytosis, exosomes, and EVs along with pathogens. STING by serving as an adapter protein recognizes cGAMP. In addition to the cGAS-derived cGAMP, MICs can uptake extracellular cGAMP expelled from cancer cell in TIME through different transporters. This induces STING ERGIC movement. ERGIC localized STING interacts with the phosphorylated IRF8 (IRF8 phosphorylation occurs as a result dsDNA recognition by cGAS/STING signaling pathway). This interaction induces STING polymerization and its interaction with TBK1 and TRAF6. The interaction between STING and TBK1 induces STING phosphorylation. The STING-bound phosphorylated TBK1 induces IRF3 phosphorylation. Phosphorylated IRF3 induces transcription of ISGs, including type 1 IFN genes. Hence, cGAS/STING/IRF8/TBK1/IRF3 axis is critical to produce type 1 IFNs and propagate type 1 IFN-mediated immune response, including the antitumor immunity. On the other hand, TBK1-mediated TRAF6 activation is critical for another canonical cGAS/STING signaling pathway involving NF-κB-dependent pro-inflammatory immune response. Thus, regulated cGAS/STING signaling critically maintains immune homeostasis and helps to clear cancer cells, whereas its dysregulation causes chronic inflammation that may lead to cancer development. Kindly see the text for details

In humans, monocytes are the primary source of type 1 IFN production via the cGAS/STING (cyclic GMP-AMP synthase (cGAS)—stimulator interferon genes (STING)) signaling pathway independent of gender [31]. cGAS/STING signaling pathway is crucial to recognize cytosolic dsDNA as a microbe or pathogen-associated molecular patterns (MAMP or PAMP) or death/damage-associated molecular pattern (DAMP) [32]. cGAS cleaves the cytosolic dsDNA into cGAMP that STING recognizes. The cGAMP-STING binding activates and recruits TANK (TRAF (TNFR-associated factor)-associated NF-κB activator)-binding kinase 1 (TBK1) and interferon regulatory factor 3 (IRF3) to the STING moved from the ER compartment to the ER-Golgi intermediate compartment (ERGIC) (Fig. 1). The STING binds with TBK1 and IRF3 to generate IRF3-dependent type 1 IFNs. Also, the STING activation stimulates the NF-κB-dependent proinflammatory cytokine release in a non-canonical manner (Fig. 1). Hence, cGAS/STING signaling-dependent type 1 IFN and NF-κB-dependent proinflammatory cytokine generation are critical in anti-infective and antitumor immunity via controlling immune surveillance and immune homeostasis (Fig. 1) [33]. The cGAS/STING signaling pathway-mediated type 1 IFN and cytokine release have been detailed elsewhere [34–38]. The cGAS/STING signaling is also crucial for antitumor effects of immune checkpoint inhibitors (ICIs), including the PD-1/PD-L1 axis inhibitors [39]. Therefore, it becomes critical to understand and discuss the role of cGAS/STING signaling in the MIC compartment of the TIME and its impact on the tumor microenvironment (TME) or TIME.

## MICs in cancer or TIME

**Fig. 2:**
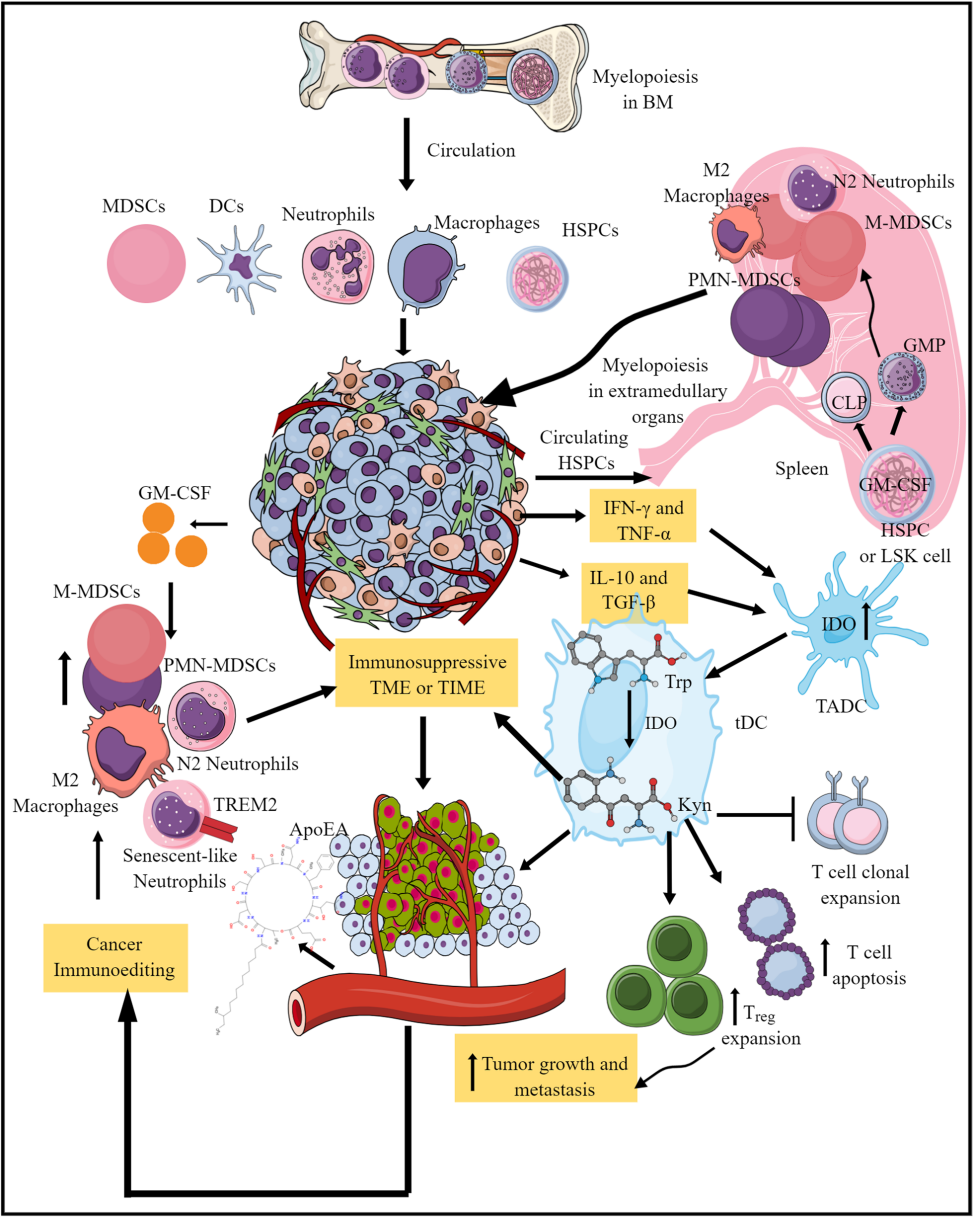
Aberrant Myelopoiesis during cancer and its impact on TIME MICs. Myelopoiesis takes place in the bone marrow under normal condition. However, cancer alters this process by inducing myelopoiesis in extramedullary organs, including spleen. During extramedullary myelopoiesis, circulating HSPCs or LSK cell move to spleen under the influence of immunosuppressive TIME. These LSK cells overexpress GM-CSF and differentiate into CLPs and GMPs. These GMPs differentiate into tumor-supportive MICs and move to the TME. GM-CSF and CCR2/CCL2 axis critically regulates the HSPC migration to the spleen for extramedullary myelopoiesis. Furthermore, GM-CSF release or increase in TIME increases immunosuppressive MICs population, including M2 macrophages, MDSCs, and N2 neutrophils that can be explained by extramedullary myelopoiesis. Tumors also exhibit cancer immunoediting to support immunosuppressive TIME. Cancer cells cleave tryptophan (Trp) to Kyn via IDO. Kyn exerts immunosuppressive activity inducing the generation of tolerogenic DCs, T_regs_ increase, and antitumor T cell apoptosis. Furthermore, pro-inflammatory (IFN-γ and TNF-α) and anti-inflammatory cytokines (IL-10, TGF-β) induce IDO overexpression in TADCs that reprograms them to tDCs secreting Kyn via increased Trp metabolism. This further increases T_reg_ development, cytotoxic T cell apoptosis, and decreases T cell clonal expansion to support immunosuppressive TIME. Thus, TIME induces or follows different mechanisms to create an immunosuppressive environment for cancer growth, development, and metastasis. Details are mentioned in the text

MICs are primary innate immune cells that target DAMPs and MAMPs/PAMPs. Macrophages, neutrophils, and DCs are the primary phagocytic cells that phagocytose apoptotic or necrotic tumor cells to enhance antitumor immunity [40–42]. Under normal conditions, myelopoiesis generates MICs in the bone marrow (BM) (Fig. 2). However, tumor-associated alterations in myelopoiesis occur in extramedullary organs that yield immunosuppressive MICs and cause subsequent tumor growth and metastasis (Fig. 2) [43–48]. The maintenance of tumor-induced immunosuppression occurs via local endogenous GM-CSF signaling that drives hematopoietic stem and progenitor cell (HSPC) myeloid commitment and differentiation into potent immunosuppressive MICs (MDSCs, M2 macrophages, and N2 neutrophils) (Fig. 2) [45–47]. The spleen of tumor bearing mice have a higher number of Lin^lo/–^Sca-1^+^c-Kit^hi^ (LSK) cells, which are early HSPCs and are highly heterogenic. For example, these LSK cells comprise different hematopoietic stem cell (HSC) and hematopoietic progenitor cell (HPC) population, which are in combined called HSPCs [45]. These LSK cells in the spleen of tumor bearing mice differentiate into FcγR^lo^CD34^+^ common myeloid progenitors (CMPs) and FcγR^hi^CD34^+^ granulocyte–macrophage progenitors (GMPs). LSK cells in the spleen of tumor bearing mice highly express GM-CSF (a critical myeloid differentiation cytokine) that upregulates NF-κB but suppresses p38 mitogen activated protein kinase (MAPK) activity. Notably, LSK cells are absent in the spleen and BM of the control animals and mice subjected to extramedullary hematopoiesis (EMH) via repeated bleeding [45]. G-CSF is not critical for LSK HSPC differentiation. Circulating HSPCs move to the spleen of tumor bearing mice for EMH for generating immunosuppressive MICs along with local naïve LSK cells, which get educated to become immunosuppressive MICs. For example, splenic stromal cells of tumor-bearing mice induce LSK cell functional alteration to generate immunosuppressive MICs via soluble factors, including IL-6. Spleens of tumor bearing mice over express CCR2 ligand CCL2, along with the CXCR2 ligands CXCL2 and CXCL5 [45]. However, CXCR2 is not expressed of HSPCs or LSK cells, instead they express CCR2. Thus, HSPCs move to spleen via GM-CSF and CCR2/CCL2 axis and where they are primed to become immunosuppressive MICs to support tumor growth and metastasis via supporting immunosuppressive TIME. Similar findings (EMH induction generating immunosuppressive MICs) have been observed in patients with different cancers [45].

Furthermore, in prostate cancer TIME, a subset of immunosuppressive neutrophils overexpress cellular senescence markers with increased persistence [49]. These senescent-like neutrophils overexpress triggering receptor expressed on myeloid cells 2 (TREM2) and are more immunosuppressive and tumor-promoting than canonical immunosuppressive neutrophils [49]. Tumor cells increase immunosuppressive senescent-like neutrophil phenotype by secreting apolipoprotein-A (ApoEA) (Fig. 2). The secreted ApoEA binds to the TREM2-expressing MICs to increase their senescence and immunosuppressive function, correlating with poor cancer prognosis (Fig. 2). Furthermore, senescent neutrophils release a great amount of exosomes carrying piRNA-17560, which induce overexpression of fat mass and obesity-associated protein (FTO) in breast cancer cells to increase the chemoresistance and epithelial to mesenchymal transition (EMT) [50]. FTO stabilizes Zinc (Zn^2+^) finger E-box-binding homeobox 1 (ZEB1) transcript and expression by lowering the N6-methyladenosine (m6A) RNA methylation to induce chemo-resistance and EMT. Therefore, the genetic deletion (SiRNA) and pharmacological inhibition (blocking ApoEA interaction with TREM2) of these senescent-like MICs, including neutrophils suppresses tumor progression in different mouse models of prostate cancers [49]. Hence, tumor-infiltrating myeloid immune cells (TIMICs) critically regulate tumor growth and metastasis [51].

Tumor-associated macrophages (TAMs) are essential in tumor immunity, progression, and cancer immunotherapy [16, 52]. TAMs influence cancer progression through M2 polarization which occurs when TAMs ingest microparticles (MPs) released by tumors in addition to the presence of IL-4, IL-13, prostaglandin E2 (PGE2), M-CSF, vascular endothelial growth factor (VEGF), hypoxia-inducible factor-1 α (HIF-1α), and high lactate levels [53–55]. The resultant alteration in their phenotypic expression promotes tumor growth, metastasis, and cancer stem cell development. Different phagocytosis-inhibiting molecules, including CD47 (integrin-associated protein or IAP) overexpression, also suppress phagocytosis-mediated innate immune defense of macrophages and DCs in the TME [56–58]. The responsiveness of cancer patients to PD-1 blockers or ICIs correlates with type 1 IFN responses in MICs. Thus, the deficiency of type 1 IFNs responsive or secretory MICs and the increase in the immunosuppressive TAMs in TIME are critical for poor cancer prognosis. For example, tumor PD-L1 (tPD-L1) engagement with MIC PD-1 (m-PD-1) activates Src homology region 2-containing protein tyrosine phosphatase 2 (SHP2) to antagonize type 1 IFN and STAT1 pathway to repress *Cxcl9* and impair CD8^+^T cell recruitment to lung metastases [59]. TIME Pro-angiogenic TAMs with diverse markers are the characteristics of different cancers [60]. A detailed timeline of TAMs and their importance at the interface of the co-evolving cancer ecosystem have been discussed in detail elsewhere [61, 62].

Notably, MDSCs are not present in healthy individuals at steady state [63, 64]. Instead, they are derived from monocytes and neutrophils exposed to chronic inflammatory conditions, including cancer, to suppress the exaggerated inflammation to prevent collateral tissue or organ damage [64]. This immunosuppressive function of MDSCs in the TIME is the primary means by which tumors evade antitumor immune responses [14, 64, 65]. Thus, MICs exert a proinflammatory antitumor immune response to remove or kill tumor cells at a premalignant stage. However, different immune evasive mechanisms, including escape from tumor immune surveillance and cancer immunoediting by tumor cells, transform these proinflammatory antitumor M1 macrophages and N1 neutrophils to tumor-supportive immunosuppressive M2 macrophages, N2 neutrophils, PMN-MDSCs, and M-MDSCs (Fig. 2) [66–69]. The detailed functional states of MICs, including classical and pathological activation states and the later stage involving MDSCs activation, have been discussed elsewhere [48]. For example, MIC-induced cancer cell lipid peroxidation (LPO) comprises an effective mechanism that governs their pathological activation stage in TIME. Neutrophil myeloperoxidase (MPO) induces tumor cell ferroptosis to create a tumor suppressive TIME for tumor growth and metastasis, including glioblastoma [70]. The neutrophil-MPO-mediated ferroptosis of cancer cells in the hyperactivated transcriptional coactivator with PDZ-binding motif-driven mouse model of glioblastoma (GBM) involves the accumulation of iron (Fe^2+^)-dependent lipid peroxides [70]. Furthermore, neutrophil and MPO-dependent GBM cell necrosis and ferroptosis have been observed in patients with GBM and predicts poor survival [70]. The intratumoral glutathione (GSH) peroxidase 4 overexpression or acyl-CoA synthetase long chain family member 4 depletion inhibits necrosis and aggressiveness of tumors. Necrosis is associated with cancer progression via angiogenesis and proliferation of endothelial cells, induces vasculature, and increases migration, invasion, and cell–cell interactions [71]. Whereas ferroptosis has a dual role in tumor pathogenesis and metastasis that depends on several factors (cytosolic and TME lactate, arginine, iron (Fe) level, Hippo signaling, intracellular and TME ROS level, cytosolic glutathione (GSH) level, and peroxisome activation) [72, 73]. Additionally, ferroptosis of immune cells comprising TIME also influences their anti or pro-tumor functions [72–74].

Furthermore, the increased metabolism of tryptophan (Trp), an essential amino acid, to kynurenine (Kyn) metabolites by indoleamine-pyrrole 2,3-dioxygenase (IDO) decreases the antigen presentation capacity of TIME DCs due to the development of tDCs (Fig. 2) [75–77]. IFN-γ (a type II IFN) is a major IDO inducer in DCs in the inflammatory environment to prevent hyperinflammatory tissue/organ damage [78–80]. IFN-γ also induces tumor-repopulating cells (TRCs) to enter dormancy by an IDO1-Kyn-aryl hydrocarbon receptor (AhR)-p27 dependent pathway in TME with TRCs expressing high levels of IDO1 and AhR [81]. The induction of the IDO1-Kyn-AhR axis by p27 prevents IFN-γ-induced STAT1 signaling-mediated tumor cell apoptosis and activates the dormancy program in TRCs [81, 82]. Thus, IFN-γ exerts antitumor and protumorigenic effects depending on the TIME [67, 82]. Additionally, TNF-α induced IDO expression promotes cancer progression (Fig. 2) [80]. The induction of tDCs restricts CD4^+^ and CD8^+^ T cell antitumor function, supporting the long-term immunosuppression in TME [76, 78, 83]. The increased Kyn metabolites and Trp depletion in the TIME also induce T cell apoptosis, T_reg_ expansion, prevent T cell clonal expansion, and the concurrent dampening of inflammatory T helper 1 (Th1)- and Th17-mediated antitumor function (Fig. 2) [76, 84]. 3-hydroxy-l-kynurenamine (3-HKA), a Kyn metabolite produced by Trp metabolism, also induces an anti-inflammatory phenotype among DCs by inhibiting IFN-γ-induced STAT1/NF-κΒ pathway-mediated TNF-α, IL-6, and IL12p70 release [84]. This further suppresses CD8^+^T cell-based antitumor immunity. As immunosuppression progresses, other immunosuppressive cytokines take over the IDO induction, including TGF-β and IL-10 (Fig. 2) [78, 85]. Notably, there are two IDO genes, IDO1 and IDO2 (much less studied and is weakly responsive to IFNs). We have discussed IDO as IDO1 here. Only some epithelial cells, DCs and macrophages express IDO, whereas lymphoid cells (T and B cells rarely express IDO 1) [85]. Interestingly, dsDNA-mediated STING activation in CD11b^+^DCs releases type 1 IFNs (IFN-α and -β), which induce IDO expression in marginal zone (MZ) CD19^+^DCs in the spleen, activating T_regs_ to promote dominant regulatory responses [86]. Interestingly, tumor draining lymph nodes (TDLNs) have an increased amount of CD19^+^ plasmacytoid DCs (CD19^+^pDCs) expressing IDO that gives them a function phenotype of tDCs, creating a local immunosuppressive TIME by suppressing the host antitumor immunity, including the T cell immunity and directly activating the mature T_regs_ [87]. Furthermore, IDO expression and activity in pDCs suppresses the T_regs_ polarization to inflammatory Th17 cells in TDLNs, which may exert antitumor immunity-depending on tumor stage and type [88, 89]. Therefore, IDO can be elevated as an early innate immune response to suppress to local inflammation in premalignant lesions that further increases at later stages of tumorigenesis associated with activated MICs and lymphoid cells [85]. However, the mechanism behind cGAS/STING and type 1 IFN-mediated IDO upregulation in MICs is yet to explore. Hence, understanding the cGAS/STING signaling pathway in TIME MICs is critical as it is controlling inflammatory and tolerogenic immunosuppressive mechanisms by controlling different mechanisms. For example, STING activation-mediated antitumor immune response works in immunogenic tumors but not in tumors with low antigenicity [85, 90].

## cGAS/STING signaling in TIME MICs

Monocytes, bridging innate and adaptive immunity, significantly affect tumor immunity, growth, angiogenesis, and metastasis [91, 92]. They exert this effect through different PRRs involved in immunity, phagocytosis of tumor cells, and releasing various cytokines, growth factors, and chemokines regulating cancer growth. cGAS/STING signaling pathway activation in monocytes also contributes to their immune function in TME or TIME (Fig. 1). There are ten known IRFs (IRF1-IRF10), which play critical roles in immunity through different mechanisms as discussed elsewhere [93–95]. Out of them, IRF8 (independent of its role in monocyte differentiation) is critical to STING-mediated innate immune responses among monocytes [96]. For example, in unstimulated monocytes/macrophages, IRF8 remains inactive by sequestering its IRF-associated domain, which prevents its interaction with STING. The cytosolic dsDNA recognition by the cGAS/STING signaling pathway phosphorylates the serine 151 of the IRF8, which initiates the interaction between the IRF-associated domain and STING (Fig. 1) [96]. The IRF8 and STING interaction is crucial for STING polymerization and TBK1-mediated STING and IRF3 phosphorylation (Fig. 1). The IRF8 overexpression in TAMs decreases tumor mass and improves patient survival in renal cell carcinoma (RCC) [97]. However, a contradictory finding suggests that IRF8 overexpression in TAMs of RCC and other cancer [kidney renal clear cell carcinoma (KIRC), lung squamous cell carcinoma (LUSC), and skin cutaneous melanoma (SKCM), breast invasive carcinoma (BRCA), bladder urothelial carcinoma (BLCA), and liver hepatocellular carcinoma (LIHC)] patients is responsible for increased cytotoxic or CD8^+^ T cell (CTL) exhaustion (PD-1 overexpression) due to increased antigen presentation [98]. The IRF8 overexpression in TAMs increases their antigen presentation potential to CTLs causing their exhaustion and worse survival outcome in ccRCC patients [98]. The IRF8-deficient TAMs are defective in producing CXCL9, a chemoattractant for CTLs in the TME or TIME [99, 100]. Thus, in addition to playing a critical role in the cGAS/STING signaling pathway, IRF8 also controls antigen presentation potential of TAMs. It will be interesting to understand the factors controlling IRF8 expression in TAMs and its function (cGAS/STING signaling activation and antigen presentation) for tumor immunotherapies to adjunct currently available ICIs.

Furthermore, cGAS/STING signaling in TAMs differs from normal macrophages by another mechanism [101]. In TAMs, protein phosphatase 2A (PP2A), with its specific B regulatory subunit Striatin 4 (STRN4), negatively regulates STING-dependent acute type I IFN production. Mice with PP2A deletion, specifically in macrophages, show reduced tumor growth and development. It is due to a decreased accumulation of immunosuppressive macrophages and increased production of type 1 IFNs with increased IFN-activated macrophages and CD8^+^T cell accumulation [101]. The STING activation involves Hippo serine/threonine kinase MST1/2 as STING agonists induce PP2A dissociation from MST1/2 in normal macrophages that are not seen in TAMs [101]. In TAMs STRN4 mediates PP2A binding to Hippo kinase MST1/2 for its dephosphorylation, stabilizing yes-associated protein/transcriptional coactivator with PDZ-binding motif (YAP/TAZ) to antagonize STING activation. For example, in human GBM patients, their TAMs overexpress YAP/TAZ, which is absent in normal macrophages [101]. Furthermore, the deficiency of PP2A/STRN4 in macrophages reduces YAP/TAZ expression and sensitizes tumor-conditioned macrophages to STING activation. Thus, PP2A/STRN4-YAP/TAZ axis is a newly explored mechanism for the immunosuppressive activity of TAMs via STING activity suppression, and its specific targeting in TAMs can sensitize tumors for immunotherapy via STING-dependent type I IFN release and increased CD8^+^T cell activity.

Although IRF8 deficiency alters DC and macrophage compartment in TIME, strategies to overexpress IRF8 in TAMs and DCs should be carefully used depending on the tumor type and their TIME. For example, many cancer types, including all desmoplastic small round cell tumors, Ewing sarcomas, synovial sarcomas, undifferentiated pleomorphic sarcomas, and all epithelial malignancies show a negative IRF8 expression [102]. ER^−^breast cancers (BCs) and triple -negative breast cancers (TNBCs) also offer little to no IRF8 expression, indicating that impaired cGAS/STING signaling supports tumor growth and metastasis due to decreased antitumor CD8^+^T cell infiltration [102, 103]. The decreased IRF8 expression in tumors (BC and pancreatic cancer) impairs the conventional DC (cDC) subset cDC1 (human CD141^+^DCs and mouse CD103^+^DCs) development, which decreases the CD8^+^T cells-based antitumor immune response [104]. The decrease in cDC1s in the absence of IRF8 can be attributed to their reprogramming to cDC2-like cells expressing CD11b and signal regulatory protein alpha (SIRPα, recognizes CD47, a “do not eat me signal” expressed on all human tumor cells), which release IL-1β and IL-6 or cDC3 cells in humans [105–107].cDC1s and their progenitors decrease systemically in cancer patients due to a decreased IRF8 expression in cDC progenitors in response to the granulocyte colony-stimulating factor (GCSF) released from local cancer cells. The increased IRF8 expression in NSCLCs increases their senescence via inhibiting AKT (protein kinase B or PKB) signaling and promoting p27 protein accumulation [108]. The uptake of dsDNA of dying senescent lung cancer cells may activate cGAS/STING signaling in TIME MICs to generate a proinflammatory antitumor immune response and the activation of CD8^+^T through increased antigen presentation. However, anti- and proinflammatory cytokine and type 1 IFN release from monocytes, along with their death and survival, depends on the concentration and binding strength of the STING ligands [109]. Thus, the low availability of STING ligands at premalignant stages may induce anti-inflammatory cytokine (IL-10 and IL-19) release. The proinflammatory cytokine release occurs due to positive regulatory (PR)/SET domain 1 (PRDI-BF1 or PRDM1) or B-lymphocyte-induced maturation protein-1 (BLIMP-1) upregulation in TIME monocytes/macrophages without their death to support tumor growth via promoting M2 polarization. Human pDCs also recognize cytosolic dsDNA via cGAS to activate STING-dependent type 1 IFNs and proinflammatory cytokines [110]. Interestingly, the STING activation in human pDCs blocks TLR9-mediated IFN production via IRF7 activation [111]. Also, STING activation in human pDCs activates the suppressor of cytokine signaling (SOCS) molecules, SOCS1 and SOCS3, that impede IFN production.

Tumor MPs (T-MPs) released from the tumor or cancer cells stimulate cGAS/STING and TBK1-STAT6 signaling pathways in TIME macrophages to reprogram them to immunosuppressive tumor-supportive M2 macrophages [53]. Also, T-MPs induce M1 macrophages’ apoptosis in the TIME or TME [53]. In patients with TNBC treated with chemotherapy or radiotherapy, T-MPs tend to be higher in PD-L1, resulting in an immunosuppressive environment and tumoral growth [112]. Furthermore, T-MPs alter M2 polarization through simultaneous TBK1 and STAT6 activation and suppression of the serine-threonine kinase (AKT)/mammalian target of rapamycin (mTOR) signaling and impair CD8^+^T cell differentiation and function due to cGAS/STING-mediated M2 macrophage polarization [112]. However, abundant cytosolic STING ligands induce the release of type I IFNs and proinflammatory cytokines (IL-1β and TNF-α) from macrophages, causing their apoptosis as indicated by a high caspase 3 (CASP3) and Poly [ADP-ribose] polymerase 1 (PARP-1) expression [109]. Hence, inducing tumor cell senescence or death (increasing IRF8 expression or radiotherapy) may increase the signal strength of cGAS/STING signaling in TIME MICs (macrophages and DCs) by phagocytosing the cytosolic dsDNA to create a proinflammatory and cytotoxic TIME. For example, STING agonist (di-amidobenzimidazole or diABZI) with radiotherapy and DNA damage repair inhibitors (DDRis) increases the survival of experimental mice with pediatric high-grade gliomas (pHGGs) [113]. We need future studies in this direction, and future therapies must consider MIC-specific STING levels and other regulatory factors to optimize immunotherapies.

## TME regulates cGAS/STING signaling in MICs via cGAMP transport

Cancer or tumor cells develop different strategies to modify infiltrated MICs, including the release of different immunoregulatory factors (cytokines, chemokines, and metabolites) [114–117]. Furthermore, cancer cells also generate different DAMPs, which modify the TIME by activating different pattern-recognition receptors (PRRs) expressed on local or infiltrated immune cells [118]. Cancer cells express different transporters, including ATP-binding cassette (ABC), lactate, glucose, and amino acid transporters through which they transport different molecule in and out to support their survival, growth, proliferation, and metastasis [119, 120]. Thus, cancer cells can also transport cGAMP to avoid endogenous STING activation as a survival and growth strategy but immune cells also express some channels or transporters, which can uptake the external cGAMP/CDN to activate internal STING-dependent type I IFN as antitumor immune function.

MDSCs are another class of immunosuppressive innate immune cells or MICs. It is interesting to understand the role of cGAS/STING signaling in their immunological function affecting TIME. cGAMP levels in TIME suppress cancer metastasis by decreasing MDSCs via STING activation, which stimulates cytotoxic antitumor CD8^+^T-cells producing IFN-γ [121]. Additionally, cGAMP-mediated STING activation in MDSCs inhibits ROS and RNS production, inhibiting immunosuppressive TIME. The ABCC1 transporter (an ATP-dependent cGAMP exporter) exports cGAMP that can be taken up by TIME immune cells, including MDSCs and macrophages [122]. The solute carrier family 19 member 1 (SLC19A1, a folate transporter) is a recently mentioned importer of cGAMP and other cyclic dinucleotides (CDNs) in MICs [123–125]. Human SLC19A1 (hSLC19A1) cavity binds two CDN or cGAMP molecules at a time for their import [124].

An alternatively spliced STING isoform known as plasmatic membrane STING (pmSTING) is localized in the plasma membrane with its C-terminus outside the cell due to the lack of one transmembrane domain in its N-terminus compared with canonical STING. The pmSTING recognizes extracellular CDNs and cGAMPs to activate STING signaling-dependent type 1 IFN and associated cytokine release to exert antitumor action (Fig. 1) [126]. Also, MICs express another cGAMP or CDN transporter called leucine-rich repeat-containing 8A (LRRC8A)/SWELL1, containing a volume-regulated anion channel (VRAC) subunit along with different tissue-specific cancer cells [127, 128]. LRRC8A forms complexes with LRRC8C and LRRC8E depending on their expression for cGAMP or CDN export and import [129, 130].

Diverse cell stress conditions (ER stress, cancer chemotherapy, and radiotherapy) activate LRRC8 channels to initiate cGAMP transport in adjacent cells, including MICs in TIME, which can stimulate the STING-dependent type 1 IFN release to exert antitumor immune function. Hence, extracellular CDNs and cGAMPs can activate STING signaling externally and through their import to MICs to generate type 1 IFNs and proinflammatory cytokines. However, not all LRRC8 VRACs mediate cGAMP; for example, LRRC8D inhibits cGAMP transport [129]. Interestingly, LRRC8A/LRRC8D heterodimer imports the anticancer drug cisplatin in cancer cells to kill them. The cGAS localized to the plasma membrane via Phosphatidylinositol 4,5-bisphosphate (PIP2) also interacts with VRACs (LRRC8A) to open them independently of its enzymatic activity [131]. The serum proteins (cGAS and TNF-α) activate LRRC8A/LRRC8E VRACs to transport cGAMP in resting cells, and heat-inactivated or proteinase K-treated serum fails to open them [131]. Thus, VRAC activation can enhance the efficacy of anticancer drugs and antitumor immunity via cGAMP transport to MICs. Notably, TME cGAMP or its administration in the TME only triggers STING activation and IFN-β production in MICs and B cells but not in NK cells [132]. The type 1 IFNs produced by MICs in response to the tumor-derived cGAMP primes NK cells to exhibit antitumor cytotoxic action [133]. For example, impaired type 1 IFN signaling in NK cells abolishes their antitumor cytotoxic function along with altering their homeostasis and development [134, 135]. The details of type 1 IFNs-mediated NK cell development, function, and homeostasis are discussed elsewhere [136, 137]. The cGAMP-induced enhancement of chimeric antigen receptor (CAR)-NK cell-based immunotherapy against pancreatic cancer has a bright future [138].

The ectopic STING expression negatively correlates with tumor-infiltrating CD33^+^ cells and decreases the percent of nasopharyngeal carcinoma (NPC)-induced HLA-DR^−^CD11b^+^CD33^+^ MDSCs [139]. Additionally, exogenous STING expression in MDSCs takes over their immunosuppressive potential as indicated by the increased proliferation of CD4^+^ and CD8^+^ T cells. Furthermore, STING inhibits MDSCs differentiation under physiological conditions [139]. CD33^+^ immature MDSCs are associated with an increased risk of cancer recurrence, such as in gastric cancer [140] and CRC [141], indicating only mature MDSCs are responsive to STING stimulus, including cGAMP. STING also influences tumor-associated MDSC induction by altering gene expression. For example, NPC STING-TBK1 signaling can upregulate the SOCS1, which interacts with the SH2 domain of the STAT3 to inhibit STAT3 phosphorylation at S727 and Y705 [139]. Notably, STAT3 is active in 70% of solid tumors, and its SH2 domain is critical in forming functional STAT3 dimers [142]. Therefore, STAT3 inhibitors are critical to explore MIC or STING-inspired cancer immunotherapies.

The antitumor effect of ICIs also depends on the antitumor activity of MIC’s cGAS/STING signaling pathway. For example, cGAS-deficient mice fail to exert the antitumor effects of the ICIs blocking PD-1/PD-L1 interaction [143]. Conversely, depleting PD-L1 in cancer cells sensitizes them to chemotherapy and the inhibitor of DNA-PK (a critical kinase for non-homologous end joining (NHEJ) pathway) [144]. Furthermore, PD-L1 depletion downregulates molecules involved in homologous recombination DNA repair pathway. Thus, DNA released from dying tumor cells can be taken up by MICs to activate the cGAS/STING pathway [144]. In human DCs, inhibiting PD-L1 can increase the antitumor action of CD8^+^T-cells in a cGAS-dependent manner against PD-L1-expressing tumor cells [145, 146]. Another checkpoint critical to cancer control is DNA damage checkpoint kinase Chk2. Changes in Chk2 expression can alter the antitumor immune response depending on the cancer type. For example, Chk2 expression levels are higher in BC than in lung cancer, indicating its more substantial effect on the cell cycle in BC [147]. In addition, chk2 checkpoint inhibition accumulates cytosolic DNA that activates the STING pathway to induce type 1 IFN and NF-κB-dependent proinflammatory antitumor immune response in AT-rich interactive domain-containing protein 1A (ARID1A)-deficient tumors [148]. Hence, MIC-mediated tumor immunity or TIME relies on active cGAS/STING signaling from its pathogenesis to antitumor effects of different chemotherapies, ICIs, and radiotherapies.

## Targeting MIC-specific cGAS/STING signaling in cancer or TME

Given that the cGAS/STING pathway is a natural immune regulator disrupted by traditional cancer therapies, modulating it through targeted therapies has excellent potential to develop adjunct therapies against cancer. Therefore, creating conditions that upregulate this pathway should reinstate tumoral IFN responses [149]. Notably, intratumoral STING activation also normalizes/reprograms tumor vasculature when administered along with VEGF receptor 2 (VEGFR2) blockers/antagonists by inducing expression of vascular stabilizing genes (e.g., angiopoietin 1 or Angpt1, platelet-derived growth factor receptor-beta or Pdgfr-β, and type IV collagen alpha protein or Col4a) [150]. Currently, we do not know about the cGAS/STING activation in MICs and its impact on angiogenesis in TME. However, cGAS/STING activation in endothelial cells is involved in the VEGF-A-dependent angiogenesis in an immune-independent manner that involves cGAS translocation to the nucleus via importin-β pathway [151]. The translocated cGAS in the nucleus regulates miR-212-5p-ARPC3 cascade to modulate VEGF-A-mediated angiogenesis by modulating cytoskeletal dynamics and VEGFR2 trafficking from trans-Golgi network (TGN) to plasma membrane through a regulatory feedback loop [151]. cGAS deficient animals show a defective VEGF-A-dependent angiogenesis, including in malignant glioma. However, it will be a novel approach to study the impact of MIC cGAS/STING signaling-dependent regulation of neoangiogenesis in the TME.

Extrinsic phagocyte (macrophages and DCs)-dependent STING signaling is crucial to dictating the immunogenicity of dying tumor cells in the TME to activate potent antitumor T cells [152]. Tumor cell escape macrophage-mediated phagocytosis via increasing the CD47 expression, which recognizes signal regulatory protein-α (SIRPα, a phagocytosis inhibitory receptor) [153]. Combining temozolomide (a chemotherapeutic agent) and CD47 blocking agents, including antagonistic anti-CD47 monoclonal antibody (mAb) increases type 1 IFN production by activating cGAS/STING signaling in myeloid antigen-presenting cells (APCs)/MICs in different cancers [154–156]. Furthermore, combining cGAMP with the antagonistic anti-CD47 mAb increases the phagocytic clearance of tumor cells and induces systemic anti-tumor immune responses, which depends on STING and type I IFN signaling [153]. STING-agonist loaded with CD47/PD-L1 targeting nanoparticles also potentiate antitumor MIC-based immunity and radiotherapy efficacy in glioblastoma [157]. CD47-SIRPα serves as an innate immune checkpoint and bridges innate and adaptive immunity in cancer [58, 158]. Tumor cells overexpress CD47 in response to the chronic DNA damage to evade immune response, including phagocytosis [159]. Notably, chemotherapy without CD47 blocking strategy enhances CD47 expression on tumor cells via inducing IL-18 release from macrophages, which increases L-amino acid transporter 2 (LAT2) in tumor cells [160]. LAT2 increases glutamine and leucine uptake in tumor cells, increasing mTORC1 signaling and Myc-dependent CD47 upregulation on tumor cells [160]. Thus, the macrophage-tumor axis is crucial in tumor immunity or TIME. Hence, chemotherapy with phagocytosis checkpoint inhibitors (PCIs) increases antitumor immunity by improving cGAS/STING signaling in MICs of TME or TIME (Table 1).

**Table 1:** Strategies used to target MIC-specific cGAS/STING signaling pathway in TIME

Therapeutic approaches in tumor type	Status (clinical or preclinical)	Specific MIC	Mechanism
Chemotherapy + PCIs in osteosarcoma [160]	Preclinical (Mouse model)	Macrophages, DCs	Increase type 1 IFN and IL-18 release and decrease LAT2 expression on tumor cells
Peptide nanotube + STING agonist (c-di-GMP-PNT) in melanoma [162]	Preclinical (Mouse model)	Macrophages	Induce STING-dependent type 1 IFN, IL-6, IL-1β and TNF-α release as well as am antitumor CD4^+^ and CD8^+^ T-cell based immune response
S100 (a STING agonist) + ICIs melanoma and colon cancer [164]	Preclinical (Mouse model)	TAMs and DCs	Type 1 IFN generation
cGAMP-VLPs in mouse melanoma and bladder cancer [165]	Preclinical (Mouse model)	cDC1s	Increases antitumor cytotoxic T cells, decreases T_regs_ and works synergistically with ICIs better than S100
Radiation + cGAMP treatment in mouse model of colorectal cancer [90]	Preclinical (Mouse model)	DCs	Type 1 IFN
Cancer vaccine (STINGVAX, a combination of CDNs and GM-CSF) in mouse model of melanoma, colon cancer, upper aerodigestive squamous cell carcinoma, and pancreatic carcinoma [171]	Preclinical (Mouse model)	DCs	Upregulate PD-L1, increase DC activation via STING activation
Radiotherapy + STING activating nano vaccine in melanoma, colon cancer and human papilloma virus-E6/E7 tumor (cervical cancer) models [143]	Preclinical (Mouse model)	Non-specific	Increase CD8^+^ T-cells in primary tumors
Cell free anticancer vaccine in mouse models of melanoma and colon cancer [179]	Preclinical (Mouse model)	DCs	Uses tumor derived MPs to transfer DNA fragments to DCs, thus activating cGAS/STING dependent acute type 1 IFN release
TTfields in GBMs [192]	Clinically approved for GBMs	DCs	cGAS/STING-dependent DCs activation induces cytotoxic T cell activation and clonal expansion
Tumor-derived exosomes as cancer vaccines, including ExoSTING, an engineered EV with CDN in mouse models of melanoma, colon cancer, and lymphoma [200]	Preclinical (Mouse model)	DCs and macrophages	Transfer dsDNA to DCs to activate type 1 IFN production and increase antitumor CD8^+^ T-cell response
STING agonist (di-ABZI) + IDO inhibitor in mouse models of colorectal cancer [201]	Preclinical (Mouse model)	DCs, MDSCs	Recruit DCs and CD8 + T-cells, inhibits MDSCs
STING agonist SHR1032 in murine colorectal cancer and acute myeloid leukemia [202]	Preclinical (Mouse model)	Macrophages, DCs	Reprogram M2 macrophages to M1, enhance antitumor function of DCs via cGAS/STING dependent signaling pathway, coordinate anticancer PARP-1 function
Mn^2+^ + ICIs mouse models of melanoma and colorectal cancer [208]	Preclinical (Mouse model)	Macrophages, DCs	Mn [2]^+^ activates cGAS/STING signaling, Promotes CD8^+^ T-cell differentiation & activation, NK cell activation, and memory CD8^+^ T-cell differentiation
Metalloimmunotherapy (ONc-Mn-A-malF127) in mouse model of colorectal cancer [210]	Preclinical (Mouse model)	DCs in TIME	Activates STING signaling in DCs, which migrate to tumor-derived lymph nodes and activate antitumor T cells to kill tumor cells
Microfabricated PLGA encapsulating cGAMP or other STING agonists in mouse model of melanoma and late-stage breast cancer [180]	Preclinical (Mouse model)	MICs in TIME along with lymphocytes	Activates STING, repolarizes M2 macrophages to M1s, induces maturation of DCs to increase their antigen presentation capacity
Zym:Ad conjugate encoding IRF3 in mouse model of melanoma [181]	Preclinical (Mouse model)	MICs in TIME	Repolarizes M2 macrophages to M1s
ProIFN or masked type 1 IFN-Fc in mouse models of melanoma, colon, and lung cancer [182, 183]	Preclinical (Mouse model)	MICs in TIME	Increases DC cross-priming, CD8^+^T cell antitumor activity

Also, high-standard human-specific cGAS small molecule inhibitors with macrophage activity have been developed with potential use in cGAS/STING signaling-mediated inflammatory diseases [161]. Recently, a peptide nanotube (PNT) loaded with the STING agonist called Cyclic diguanylate monophosphate (c-di-GMP) has been developed, which has limited efficacy due to its poor membrane permeability and low bioavailability [162]. The c-di-GMP-PNT treatment's nanocomposite induces a STING-dependent type 1 IFN, IL-6, IL-1β, and TNF-α release from macrophages, initiating a potent antitumor CD4^+^ and CD8^+^T cells-based immune response (Table 1). Further studies have shown the efficacy of STING agonism in non-tumor cells and MICs in poorly immunogenic tumors, including the B16F10 melanoma model [163]. Another STING agonist called ADU-S100 (S100), in combination with ICIs, increases survival and durable protection in a poorly immunogenic tumor through type 1 IFN generation-dependent manner, independent of TNF-α generation (Table 1) [164]. A more robust and effective technique than S100 has been developed recently to selectively induce the DC-specific STING activation in the TIME for priming antitumor T cell response [165]. The technique incorporates cGAMP with non-infectious virus-like particles (VLPs, cGAMP-VLPs) that specifically activates TIME cDC1s STING signaling pathway upon intratumoral injection (Table 1). The cDC1 STING signaling activation in the TIME with cGAMP-VLPs at a 1000-fold lower dose than S100 increases the differentiation of circulating antitumor T cells, decreases T_regs_ in the TIME, and works synergistically with PD-1 blockers (Table 1) [165]. It is important to note that S100 eliminates TIME cDC1s but cGAMP-VLPs act on cDC1’s STING to exert antitumor action.

In the immunogenic MC38 model of murine colon cancer (CC), vital STING signaling in tumor cells can partially bypass the requirement for STING-dependent activity from immune cells, including MICs. Notably, cGAS/STING signaling is crucial for the efficacy of ICIs in different tumors [39, 163]. Also, radiation-induced type 1 IFN-dependent antitumor immunity in immunogenic tumors depends on detecting cytosolic DNA by the STING signaling in TIME DCs [90]. Additionally, the exogenous cGAMP induction in the TIME promotes the efficacy of radiation therapy. However, the non-canonical NF-κB activation downstream to cGAS/STING signaling pathway in tumor-associated DCs (TADCs) during radiation therapy decreases type 1 IFNs level [166]. Thus, explicitly inhibiting non-canonical NF-κB activation downstream to cGAS/STING signaling during radiation therapy in TADCs enhances the antitumor efficacy of radiation therapy.

Cancer vaccines can utilize STING to improve their efficacy in PD-1-resistant cancers. Notably, nuclear PD-L1 silencing increases STING promotor activity. Specifically, PD-L1 binds to the STING promotor region, thus being a regulatory factor in STING expression and cancer growth [167]. STINGVAX, a cancer vaccine comprising CDNs (activating TBK1/IRF3, NF-κB, and STAT6 signaling pathways) and GM-CSF, has shown promising results in different murine tumor models (Table 1). Notably, GM-CSF use in cancer needs a caution due to its controversial role in tumorigenesis and antitumor activity [168–170]. Specifically, mice treated with STINGVAX upregulate PD-L1 and increase DC activation (Table 1) [171]. DCs uptake nanoparticles (NPs) from the vaccine and activate the STING pathway [143]. Cancer therapies (cGAMP treatment) via cGAS/STING pathway reprogram MICs (M2 to M1 macrophages or N2 to N1 neutrophils) to inhibit tumoral advancement [172–175]. Infusions with ONP-302 activate the cGAS/STING pathway in MICs, activating NK cells and increasing PD-1/PD-L1 expression in the TIME [176]. STING-targeting vaccines can be administered to supplement traditional therapies to enhance the antitumor response. For example, co-treatment with radiotherapy and a STING activating nano vaccine enhance tumoral response compared to either treatment independently. Specifically, treating established murine tumors in this manner increases the number of antitumor CD8^+^ T cells in primary tumors [177].

Cancer vaccines also activate the antitumoral properties of the cGAS/STING pathway utilizing tumor-derived MPs and DNA signaling. For example, MPs can facilitate antigen transfer from macrophages to DCs, thus serving a differential role in cancer immunity [178]. Specifically, cell-free anticancer vaccines can utilize tumor-derived MPs to transfer DNA fragments to DCs. Furthermore, the cytosolic DNA in DCs triggers cGAS/STING signaling-dependent type 1 IFN release, further supporting DC maturation and tumor antigen presentation to CD4^+^ and CD8^+^ T cells [179]. In addition, engineered cancer therapies can utilize the STING pathway to overcome challenges associated with traditional cancer vaccines. For example, therapeutics developed with microfabricated polylactic-co-glycolic acid (PLGA) particles stay at the injection site and release STING agonists at pre-programmed time points through pulses, mimicking multiple injections. This technique seems promising in different cancers in mice in terms of inducing a robust antitumor immune response inhibiting tumor growth and metastasis to prolonged survival (Table 1) [180]. The microfabricated PLGA particles encapsulating the STING agonist stay at the injection site and release STING agonists at pre-programmed time points through pulses that mimic injections. This technique has shown promise in multiple mouse cancers by inhibiting tumor growth, reducing metastasis, and prolonging survival [180]. The STING agonist encapsulated in the microfabricated PLGA is not MIC-specific, but modulates CD4^+^, CD8^+^, and NK cells (increased antitumor action) along with repolarizing M2 macrophages to M1 macrophages and inducing DC maturation to serve as potent APCs to activate antitumor T cells (Table 1) [180]. Also, zymosan: adenovirus (Zym:Ad) conjugate encoding IRF3 repolarizes M2 macrophages to antitumor M1 macrophages in the TME and increases systemic antitumor immunity (Table 1) [181].

Interestingly, researchers have designed a masked type I IFN-Fc (ProIFN) with its natural receptor connected by a cleavable linker, an easy target of tumor-associated proteases [182]. ProIFN has a high serum half-life, improved tumor targeting via an enhanced DC cross-priming, and significantly increased CD8^+^T cell infiltration and effector function in the TME. Furthermore, these type 1 IFNs induce granzyme B (Gzm-B) expression in CD8^+^T cells by STAT3 activation to increase their cytotoxic action against tumor cells [183]. However, type 1 IFNs may protect tumors expressing IFN-αR1 via *Serpinb9*, an IFN-inducible gene encoding Serpin B9 or proteinase inhibitor-9 (PI-9, a serine protease), an endogenous Gzm-B inhibitor against ionizing radiation therapy [184, 185]. Thus, blocking IFN-αR on tumor cells or an adjunct treatment with Serpin B9 inhibitor (anti-Serpin B9 therapy) can increase cytotoxic CD8^+^T cells against tumor cells and ICI's antitumor action [186, 187]. Hence, these approaches are helpful in tumors where a direct cGAS/STING signaling pathway activation cannot produce type 1 IFNs.

The systemic or direct intratumoral CDN or STING activators help tumor clearance in preclinical models but fail to induce antitumor immune memory [188–191]. On the other hand, tumor treating fields (TTFields), an approved therapy for GBM and malignant mesothelioma, induces cGAS/STING signaling and absent in melanoma-2 (AIM-2)-dependent type 1 IFN and proinflammatory cytokine-based antitumor immunity in syngeneic mouse GBM model developed using KR158 and GL261 glioma cells (Table 1) [192]. TTFields-treated GBM cells also generate antitumor immune memory via STING- and AIM2-dependent signaling pathways in DCs to promoting cytotoxic T cell activation and clonal expansion (Table 1) [192, 193]. Notably, robust post-TTFields activation of adaptive immunity in patients with GBM via a type 1 IFN-based trajectory has been reported. Still, the authors have not indicated the STING activation in the GBM cells. Hence, TTFields in GBM patients work by stimulating specifically cGAS/STING in MICs but not in GBM cells as they have methylated STING. Therefore, TTFields and DNMT combination can be a better treatment approach for GBM patients.

Extracellular vesicles (EVs) are crucial in carcinogenesis and metastasis [194] and should be explored in context of cancer vaccines and the cGAS/STING pathway among MICs. Modified EVs can deliver therapeutic agents such as chemotherapies or nucleic acids with high efficiency and specificity for cancer vaccines [195]. EVs are prevalent in the TIME and play a key role in cancer advancement [194]. For example, TNBCs release EVs, promoting monocyte differentiation to proinflammatory macrophages, causing increased T cell infiltration and an improved prognosis [196] A subset of EVs known as exosomes is an exciting avenue to explore concerning cancer vaccines, as they are secreted by nearly all cell types and facilitate communication between innate immune and invading cells to regulate innate immunity [197]. Irradiated, but not unmodified, tumor-derived exosomes have already shown promise as a cancer vaccine in mice. Tumor-derived exosomes in irradiated mouse BC cells can transfer dsDNA to DCs, thus activating DCs and STING for type 1 IFN production (Table 1). The irradiated tumor-derived exosomes also increase antitumor CD8^+^T cell responses in vivo [198]. Additionally, EVs derived from activated CD4^+^T cells (T-EVs) carrying IFN-γ sensitize TAMs via STING activation (without cGAS and cGAMP involvement) to repolarize them to antitumor M1 macrophages in TIME [199]. Hence, T-EVs are another option to repolarize immunosuppressive TAMs to antitumor M1 macrophages to enhance the antitumoral function of cGAMP.

Recently ExoSTING, engineered EVs loaded with external CDN, has increased CDN potency to elicit a potent antitumor immune response via activating APCs (Table 1) [200]. Intratumoral ExoSTING stays within the TME and enhances CD4^+^T cell-dependent Th1 immunity and CD8^+^T cell-dependent cytotoxicity, generating systemic antitumor immunity. However, ExoSTING does not induce a systemic proinflammatory immune response at therapeutic doses, leaving an enhanced therapeutic window. Further research is warranted into how EV-based STING agonists and vaccines can advance cancer immunotherapy. Additionally, a STING agonist (di-ABZI) and IDO inhibitor (1-methyl-d-tryptophan or 1-MT) combination inhibits tumor progression in the murine CRC via recruiting CD8^+^T cells and DCs and inhibiting immunosuppressive MDSCs (Table 1) [201]. Another STING agonist, SHR1032, is tumor protective in murine CRC by stimulating the type 1 IFNs, TNF-α, and IL-6 release in the TME (Table 1) [202]. Additionally, STING agonism in breast cancer gene-1 (BRCA-1) deficient BC mouse model reprograms M2 macrophages to antitumor M1 macrophages, enhances the antitumor function of DCs, and synergizes the anticancer effects of PARP-inhibitors (PARP-Is) [203, 204].

MICs are critical to STING-based cancer therapy at the TIME. STING activating therapies can suppress MICs in humans and mice by inhibiting Myc signaling and altering metabolic processes and cell cycle. These anticancer responses are due, in part, to MIC repolarization and T-cell activation and are strengthened when injected directly into the tumor [205]. This antitumor effect involves Myc inactivation, which was previously difficult to achieve. Manganese (II) ions (Mn^2+^) promote DC and macrophage maturation and tumor-specific antigen presentation, potentiate CD8^+^T cell differentiation, activation, NK cell activation, and memory CD8^+^T cell differentiation. Combining Mn^2+^ with ICIs synergistically boosts antitumor immunity and decreases ICIs dose [206]. In addition, Mn^2+^ activates the cGAS/STING signaling pathway via activating the TBK1 phosphorylation for immunomodulation (Table 1) [207]. Therefore Mn2^+^ has been used in several ways to target cancer. For example, the cancer metalloimmunotherapy comprising CDN STING agonists and Mn^2+^, assembled into an NP called CDN-Mn^2+^ particle or CMP induces antitumor immunity in many murine tumor models [208].

Furthermore, bovine serum albumin (BSA)/ferritin-based nanoagonist incorporating Mn^2+^ amplifies cGAS/STING signaling, and β-lapachone activates cGAS/STING signaling in DCs to elicit robust T cell-dependent adaptive antitumor immunity [209]. Additionally, Mn^2+^ coordination micelle (ABZI (STING agonist) + naphthalocyanine (ONc) in maleimide-modified Pluronic F127 (malF127) or ONc-Mn-A-malF127) also forms a novel cancer metalloimmunotherapy to stimulate STING in the TME via capturing in situ tumor antigen (Table 1) [210]. Thus, ONc-Mn-A-malF127 creates a nanoplatform for increasing the efficacy of anticancer therapy with metalloimmunotherapy and photo-induced immunogenic cell death (ICD)-based immunotherapy with an abscopal solid effect.

The biodegradable STING agonists comprising poly (beta-amino ester) (PBAE) NPs loaded with CDNs have decreased the amount of CDN needed to stimulate STING for potent antitumor immunity in combination with PD-1 blockers [211]. Furthermore, STING-NPs increase the cGAMP's potency to activate the STING-dependent antitumor immunity in the TME and lymph nodes (LNs) in different cancers [212]. Also, cGAMP-NPs stimulate STING more strongly than cytosolic cGAMP, reprogram M2 macrophages to antitumor M1 macrophages, and increase IFN-γ producing antitumor T cells [213, 214]. The extracellular matrix (ECM)-degrading nanoagonist (dNAc) with a second near-infrared (NIR-II) light-controlled system also activates STING-dependent type 1 IFN release during mild photothermal-augmented chemodynamic-immunotherapy in a mouse model of breast cancer [215]. The dNAc, upon NIR-II photoactivation, releases different immunogenic tumor antigens, including dsDNA activating tumoral DCs to stimulate antitumor T cell immunity through STING activation. In addition, the STING activation in DCs upregulates IL-15Rα expression in type 1 IFN-dependent manner, increasing NK cell number and their antitumor function [216, 217]. Hence, cGAS/STING signaling in DCs activates NK and T cell-mediated antitumor immunity. Furthermore, another study has shown that the targeted STING activation in APCs (macrophages and DCs) in the TME under spatiotemporal ultrasound stimulation increases systemic antitumor immunity and improves the ICI's therapeutic efficacy [218]. Thus, MIC-based cGAS/STING signaling targeting is a novel immune-based approach to target tumors and increase antitumor immunity.

## Future perspective and conclusion

Tumor cell and MIC-specific cGAS/STING activation are essential for inducing an initial antitumor immune response. However, the chronic cGAS/STING signaling pathway activation in tumors induces immunosuppressive TIME and cancer cell survival and metastasis. Furthermore, radiotherapy alone enhances the immunosuppressive action of MDSCs via DNA damage-induced cGAS/STING signaling after its cessation through enhancing suppressive inflammation in tumors by recruiting myeloid cells (M-MDSCs) in part via chemokine receptor 2 (CCR2, a receptor for CCL2, CCL7, and CCL12) signaling pathway resulting tumor radioresistance [219, 220]. However, STING activation in tumor MDSCs in response to cytosolic mitochondrial DNA (mtDNA) restores their immunostimulatory function by releasing antitumor type 1 IFNs and proinflammatory cytokines [221]. The pancreatic ER kinase (PKR)-like endoplasmic reticulum (ER) kinase (PERK) expression in MDSCs in TME or TIME via nuclear factor erythroid 2–related factor 2 (NRF2) activity-mediated mitochondrial homeostasis determines the STING signaling-dependent antitumor type 1 IFNs release [222]. Notably, the PERK inhibition in cancer cells induces parapotosis that stimulates type I IFN production in response to released ATP and HMGB1 in DCs independently of STING activation.

Conversely, the STING activation primes CCR2-dependent tumor trafficking of common-monocytic precursors and their intra-tumor commitment into monocytic-lineage inflammatory Ly6C^+^CD103^+^ DCs [223]. Although human T cells express high STING levels and in the presence of TCR-engage signaling, cGAS/STING signaling activation switches T cells to produce type 1 IFN. Still, STING activation/agonism impedes their antitumor function [224]. This indicates MIC-specific cGAS/STING targeting is more crucial for developing tumor immunotherapy than T cell-specific one.

We should be careful using systemic STING agonist monotherapy in different cancers as STING activation alone, as a tumor therapy, has indicated the emergence of tumor resistance in clinical trials [225]. The development of tumor resistance with the systemic STING agonist (cGAMP) monotherapy involves IL-35 released by B cells (regulatory B cells or B_regs_) in an IRF-3-dependent manner, suppressing antitumor NK cells in the TME [226, 227]. The STING-dependent tumor resistance can be overcome by combining the STING agonist CDN with an IL-2 superkine (half-life-extended IL-2) and H9-mouse serum albumin (H9-MSA) or CDN/H9-MSA against resistant tumors, which are either MHC 1-deficient or positive [228]. The CDN/H9-MSA approach has induced a more potent and prolonged NK cell-dependent antitumor immune response than STING agonist-based monotherapy, causing a long-term remission from cancer in different animal models.

Tumors should be checked for clathrin-associated adaptor protein complex-1 (AP-1) levels in TME and associated MICs as it controls STING signaling termination [229]. For example, AP-1 sorts phosphorylated STING into clathrin-coated transport vesicles for the endolysosomal system delivery to degrade it by recognizing the cytosolic STING CTT [229, 230]. Hence, it will aid in research to understand the impaired cGAS/STING signaling in the TME and MICs. Also, in human MICs, the cGAS/STING-mediated cytosolic DNA detection induces their death by increased potassium (K^+^) efflux [231]. This K^+^ efflux occurs in response to the transport of activated STING to the lysosome, causing membrane destabilization and lysosomal cell death (LCD).

Furthermore, the increased K^+^ induces NLPR3 activation and IL-1β release associated with MIC death [231, 232]. It will be interesting to observe this finding in TIME. Also, transmembrane protein 203 (TMEM203) is a binding partner of STING and regulates its downstream proinflammatory signaling in macrophages upon cGAMP recognition [233]. Hence, it will be novel to investigate the role of TMEM203 in TIME macrophages of different solid cancers as it regulates intracellular Ca^2+^ levels, which is crucial in apoptosis [234, 235].

Furthermore, the loss of Golgi-to-lysosome STING cofactors, but not ER-to-Golgi cofactors, selectively activates the tonic IFN signaling in cells [236]. For example, post-Golgi trafficking impairment extends STING Golgi-dwell time to increase type 1 IFN-dependent immune signaling. However, its prolonged increase may create a chronic inflammation predisposing to cancer development. Trans-Golgi coiled-coil protein GCC2 and several Rab GTPases are key regulators of post-Golgi trafficking of the STING. Thus, investigating GCC2 and Rab GTPases in TIME MICs defective in STING-dependent antitumor IFNs and cytokine production will help develop new cell-specific cGAS/STING signaling targeting therapeutics. For example, cancer cells without GCC2 and Rab14 induce T-cell and IFN-dependent antitumor immunity and inhibit tumor growth in vivo.

On the other hand, STING agonist DMXXA exerts antitumor effects by decreasing tumor vessel size and increasing the proportion of tumor-specific T cells in the TME  [237]. However, this effect was reduced by anti-CD40 antibodies or IL-2-based immunotherapies. A recent study has indicated the activation of CD11b integrin (comprised of integrin αM (ITGAM) and β2 (CD18) integrins) on TAMs in pancreatic ductal adenocarcinoma (PDAC) via its agonist called GB1275 (LA1 or ADH503) activates STING and STAT1-dependent increased type 1 IFN and CXCL9*, *10*,* and 11*,* but decreased IL-1α and IL-1β release [238]. The GB1275-mediated CD11b targeting in TAMs also induces CD8^+^ T cells to their proliferative effector phenotypes that further enhances the antitumor activity. Notably, CD11b activation in TAMs inhibits NF-κB activation and decreases NF-κB/IL-1 gene signature by ubiquitination-mediated p65 degradation to alter their protumor phenotype to antitumor [238]. The CD11b stimulation in TAMs activates STING-dependent type 1 IFN generation by an axis called focal adhesion kinase (FAK)/sirtuin-3 (SIRT3)/ reactive oxygen species (ROS). The ROS over production induces mitochondrial damage that releases mitochondrial DNA (mtDNA) in the cytosol to activate cGAS/STING signaling and STAT1 dependent antitumor immunity. Thus, different new regulatory pathways are coming up to control cGAS/STING signaling pathway-dependent immune responses in MICs. Hence, cGAS/STING signaling in MICs is critical to understand different solid tumors' TIME, and STING agonists should be used cautiously with other immunotherapies.

## Data Availability

Not applicable.

## Abbreviations

APC: Antigen presenting cell
ARID1A: AT-rich interactive domain-containing protein 1A
BC: Breast cancer
BLIMP-1: B-lymphocyte-induced maturation protein-1
BM: Bone marrow
BrM: Brain metastasis
c-di-GMP: Cyclic diguanylate monophosphate
cDC: conventional Dendritic cell
CDN: Cyclic dinucleotide
CMP: Common myeloid progenitor
DAMP: Death/damage-associated molecular pattern
DDRis: DNA damage repair inhibitors
dNAc: Degrading nanoagonist
EMH: Extramedullary hematopoiesis
EMT: Epithelial to mesenchymal transition
ERGIC: ER-Golgi intermediate compartment
FTO: Fat mass and obesity-associated protein
G-CSF: Granulocyte colony-stimulating factor
GBM: Glioblastoma
GM-CSF: Granulocyte monocyte-colony-stimulating factor
GMP: Granulocyte–macrophage progenitor
HIF: Hypoxia-inducible factor
HSC: Hematopoietic stem cell
HSPC: Hematopoietic stem and progenitor cell
ICI: Immune checkpoint inhibitor
IRF: Interferon regulatory factor
ITGAM: Integrin αM
LN: Lymph node
LPO: Lipid peroxidation
LUAD: Lung adenocarcinoma
MAMP: Microbe-associated molecular patterns
MDSC: Myeloid-derived suppressor cell
MICs: Myeloid immune cells
MPO: Myeloperoxidase
MP: Microparticle
mtROS: Mitochondrial reactive oxygen species
mtDNA: Mitochondrial DNA
NPC: Nasopharyngeal carcinoma
NPs: Nanoparticles
NRF2: Nuclear factor erythroid 2-related factor 2
NSCLC: Non-small cell lung cancer
PAMP: Pathogen-associated molecular pattern
PARP-1: Poly [ADP-ribose] polymerase 1
pDC: Plasmacytoid dendritic cell
PDAC: Pancreatic ductal adenocarcinoma
PERK: Pancreatic ER kinase (PKR)-like endoplasmic reticulum (ER) kinase
pHGG: Pediatric high-grade glioma
PICs: Phagocytosis checkpoint inhibitors
PIP2: Phosphatidylinositol 4,5-bisphosphate
PLGA: Polylactic-*co*-glycolic acid
pmSTING: Plasmatic membrane STING
PNT: Peptide nanotube
PP2A: Protein phosphatase 2A
PR: Positive regulatory
PRDM-1: Positive regulatory (PR)/SET domain 1
PRRs: Pattern recognition receptors
RCC: Renal cell carcinoma
RNS: Reactive nitrogen species
ROS: Reactive oxygen species
SIRPα: Signal regulatory protein alpha
SIRT-3: Sirtuin-3
SOCS: Suppressor of cytokine signaling
STAT-3: Signal transducer and activator of transcription-3
STRN4: Subunit striatin 4
TAM: Tumor-associated macrophages
TAZ: Transcriptional coactivator with PDZ-binding motif
TBK: TANK binding kinase
TCR: T-cell receptor
tDCs: Tolerogenic dendritic cells
TF: Transcription factor
TIME: Tumor immune microenvironment
TIMIC: Tumor infiltrating myeloid immune cell
TME: Tumor microenvironment
T-MP: Tumor microparticle
TNBC: Triple negative breast cancer
TRCs: Tumor repopulating cells
TTFields: Tumor treating fields
VRAC: Volume-regulated anion subchannel
YAP: Yes-associated protein
VEGF: Vascular endothelial growth factor
